# Negative association of the chemokine receptor CCR5 d32 polymorphism with systemic inflammatory response, extra-articular symptoms and joint erosion in rheumatoid arthritis

**DOI:** 10.1186/ar2733

**Published:** 2009-06-18

**Authors:** Manuela Rossol, Matthias Pierer, Sybille Arnold, Gernot Keyßer, Harald Burkhardt, Christoph Baerwald, Ulf Wagner

**Affiliations:** 1 Division of Rheumatology, Department of Internal Medicine II, University of Leipzig, Johannisallee 30, 04103 Leipzig, Germany; 2 Department of Internal Medicine I, University of Halle/Saale, Ernst-Grube-Straße 40, 06120 Halle/Saale, Germany; 3 Division of Rheumatology, Department of Internal Medicine II, Johann Wolfgang Goethe University Frankfurt am Main, Theodor-Stern-Kai 7, 60590 Frankfurt am Main, Germany

## Abstract

**Introduction:**

Chemokines and their receptors control immune cell migration during infections as well as in autoimmune responses. A 32 bp deletion in the gene of the chemokine receptor CCR5 confers protection against HIV infection, but has also been reported to decrease susceptibility to rheumatoid arthritis (RA). The influence of this deletion variant on the clinical course of this autoimmune disease was investigated.

**Methods:**

Genotyping for CCR5d32 was performed by PCR and subsequent electrophoretic fragment length determination. For the clinical analysis, the following extra-articular manifestations of RA were documented by the rheumatologist following the patient: presence of rheumatoid nodules, major organ vasculitis, pulmonary fibrosis, serositis or a Raynaud's syndrome. All documented CRP levels were analyzed retrospectively, and the last available hand and feet radiographs were analyzed with regards to the presence or absence of erosive disease.

**Results:**

Analysis of the CCR5 polymorphism in 503 RA patients and in 459 age-matched healthy controls revealed a significantly decreased disease susceptibility for carriers of the CCR5d32 deletion (Odds ratio 0.67, *P *= 0.0437). Within the RA patient cohort, CCR5d32 was significantly less frequent in patients with extra-articular manifestations compared with those with limited, articular disease (13.2% versus 22.8%, *P *= 0.0374). In addition, the deletion was associated with significantly lower average CRP levels over time (median 8.85 vs. median 14.1, *P *= 0.0041) and had a protective effect against the development of erosive disease (OR = 0.40, *P *= 0.0047). Intriguingly, homozygosity for the RA associated DNASE2 -1066 G allele had an additive effect on the disease susceptibility conferred by the wt allele of CCR5 (OR = 2.24, *P *= 0.0051 for carrier of both RA associated alleles)

**Conclusions:**

The presence of CCR5d32 significantly influenced disease susceptibility to and clinical course of RA in a German study population. The protective effect of this deletion, which has been described to lead to a decreased receptor expression in heterozygous patients, underlines the importance of chemokines in the pathogenesis of RA.

## Introduction

Chemokines are chemoattractant cytokines, which play a central role in T cell migration to and infiltration into the synovitic lesions in joints of patients with rheumatoid arthritis (RA). The CC chemokines RANTES, MIP-1α, MIP-1β, and MCP-1 are strongly expressed in the synovial membrane of patients with RA, and the primary CC chemokine receptor found on T cells in rheumatoid synovium is CCR5 [1]. In addition, CCR5 is expressed on tissue macrophages and on a high proportion of T cells and natural killer (NK) cells in synovial fluid, while only a small subpopulation of peripheral blood monocytes is CCR5 positive [2].

A 32 bp deletion in the CCR5 gene, termed CCR5d32, results in a frame shift and a nonfunctional receptor, and homozygosity for CCR5d32 has been shown to prevent transmission of HIV-1, while heterozygosity prolongs the time period between infection and the development of AIDS [3,4].

This deletion has also been found to be protective against the development of RA [5-7], although the results remain somewhat controversial [8]. The gene copy number of chemokine-ligand-3 like-1 (CCL3L1), a ligand for CCR5, has also been found to be associated with susceptibility to RA [9].

In association studies with other autoimmune diseases, a significant protective effect of the deletion against more severe clinical courses of multiple sclerosis [10], systemic lupus erythematodes [11], Crohn's disease [12], primary Sjögren's disease [13], Behçet's disease [14], and lung disease in sarcoidosis [15] was observed. More recently, associations with CCR5d32 have also been described for primary sclerosing cholangitis [16], cardiovascular disease [17], and juvenile idiopathic arthritis [18].

In addition to its impact on disease susceptibility, the CCR5d32 deletion has been shown to influence the clinical course of RA. Patients carrying the CCR5d32 deletion were found to be more frequently negative for rheumatoid factor (RF) IgM and to have fewer swollen joints and a shorter period of morning stiffness [19] and more frequently have a non-severe course of RA [20], but results remain conflicting [6].

The goal of our study was, therefore, to investigate the influence of the CCR5d32 deletion on disease susceptibility and on the clinical course of RA in a large and clinically well characterized German patient cohort, which has previously been analyzed for other genetic influences [21-23].

## Materials and methods

### Patients and controls

Five hundred and three patients with RA according to the 1987 revised criteria of the American College of Rheumatology were included in the study. The study design was approved by the University of Leipzig's ethics committee, and informed consent was obtained from each patient before study enrolment. Characteristics of the patient cohort are displayed in Table 1.

**Table 1:** Characteristics of the rheumatoid arthritis patient cohort

Number of patients (female/male)	503 (369/134)
Age at onset (years) (median (range))	49 (18 to 84)
Disease duration (years) (median (range))	16 (2 to 70)
Patients positive for RF IgM (%)	76.9
Patients positive for RF IgA (%)	53.2
Patients positive for anti-CCP antibodies (%)	70.5
Patients positive for ANA (%)	36.9
Extra-articular manifestations	
Rheumatoid nodules (%)	31.4
Polyserositis (%)	2.4
Interstitial pulmonary fibrosis (%)	5.5
Raynaud's syndrome (%)	5.2
Vasculitis (%)	12.5
Erosive disease (%)	80.7

ANA = Antinuclear antibodies; CCP = cyclic citrullinated peptide; RF = rheumatoid factor.

The presence of erosive joint disease was evaluated by analyzing the previously available hand and feet radiographs of the patients. The presence of extra-articular manifestations of the disease was judged by retrospective chart review and by analysis of an available clinical database as described previously [24]. The following extra-articular manifestations of the disease were documented by the rheumatologist following the patient: presence of rheumatoid nodules, major organ vasculitis, pulmonary fibrosis, serositis, or a Raynaud's syndrome. Also by retrospective chart review, all available C-reactive protein (CRP) values from 359 patients were entered into a data base, and the median value was calculated and used for statistical analysis.

RA cases from two separate studies were enrolled for the replication study. One-hundred and eight-two patients had been part of a clinical study evaluating the influence of genetic parameters on the progression of joint destruction [21,25] and 291 cases from an inception cohort of early RA patients were enrolled, which has also been published previously [21,26,27]. For both cohorts, radiographic data from hand and feet radiographs were available, which had been scored according to the Ratingen score [28]. Radiographs in the early RA cohort were taken at study entry and subsequently after two years of observation. In the retrospective study, the last available radiograph taken during the first 10 years of disease duration was analyzed (n = 158). In addition, in 118 patients, a radiograph taken after more than 10 years of disease duration was available and was analyzed separately.

In addition, the CRP level at onset of disease as determined at initial presentation with a rheumatologist was available for analysis for the inception cohort of patients with early RA. Data on extra-articular manifestations were not available in both cohorts.

From among healthy blood donors with ethics committee approval 459 age-matched control subjects with no history of inflammatory arthritis were recruited. Controls and RA patients were Caucasian subjects of German origin with no discernable ethnic variation.

### Genotyping methods

Genomic DNA was isolated from 10 ml of peripheral blood using standard procedures and amplified by PCR. The following oligonucleotide primers were used to detect CCR5 d32: sense 5'-TTT ACC AGA TCT CAA AAA GAA G and anti-sense 5'-GGA GAA GGA CAA TGT TGT AGG [2]. Reaction mixtures (25 μl) contained DNA (100 to 200 ng) and oligonucleotide primers (20 pM).

The mixture was heated at 94°C for three minutes and then subjected to 40 amplification cycles of 94°C for 30 seconds, 62°C for one minute and 72°C for one minute, followed by a final elongation cycle of 72°C for five minutes. The resulting PCR products, 274 bp for CCR5 wildtype and 242 bp for CCR5 d32, were separated on an ethidium bromide stained 2% agarose gel by electrophoresis and visualized by ultraviolet light.

### Detection of autoantibodies

The presence of RF was determined by laser nephelometry according to the manufactor's instructions (Dade Behring, Liederbach, Germany). Individuals with values of 40 IU/ml on at least one occasion were counted as RF positive.

For the detection of anti-cyclic citrullinated peptide (CCP) antibodies in patient sera, a commercially available, second generation anti-CCP ELISA (Immunscan RA2, Generic Assays, Dahlewitz, Germany) was used. A cut off of 25 U/ml was used as a stringent criterion for anti-CCP antibody positivity.

Antinuclear antibodies (ANA) were detected using immunofluoresence and a cut-off titer of 1:320 was used as a stringent criterion for ANA positivity.

### Statistical analysis

Allele and genotype frequencies of CCR5 d32 were obtained by direct counting. For allele and genotyping comparisons, the chi-squared test with 2 × 2 contigency tables (alleles) or 2 × 3 contigency tables (genotypes) was used. Odds ratios (OR) and 95% confidence intervals (CI) were calculated according to Woolf's method. For the analysis of an interaction between DNASE2 SNP alleles and CCR5d32 in conferring disease susceptibility to RA, McNemar's test was used. *P *values of less than 0.05 were considered statistically significant. The software used was the Sigmastat program (Systat 2004, Richmond, CA, USA).

## Results

The distribution of genotypes and the observed allele frequencies of the CCR5d32 deletion in healthy controls and RA patients are shown in Table 2. The CCR5 dd32 deletion was present less frequently in RA patients compared with controls (allele frequency 10.0% versus 13.4%, *P *= 0.0262). Accordingly, the wild type allele at CCR5 d32 conferred an OR of 1.39 (95% CI = 1.05 to 1.84) for developing RA.

**Table 2:** CCR5 d32 case-control analysis

		Controls (n = 459)	RA (n = 503)	*P *values
Allele	wt	795 (86.6)	905 (90.0)	
	d32	123 (13.4)	101 (10.0)	0.0262
				
genotype	wt/wt	343 (74.7)	409 (81.3)	
	wt/d32	109 (23.7)	87 (17.3)	
	d32/d32	7 (1.5)	7 (1.4)	0.0437
				
	wt/d32+ d32/d32	116 (25.3)	94 (18.7)	0.0168

Values are the absolute numbers of genotypes or alleles with frequencies in percent given in parentheses. *P *values were calculated using chi-squares test with 2 × 2 contigency tables (alleles) or 2 × 3 contigency tables (genotypes) for comparison of frequencies of the indicated markers in the patients compared with the controls.

RA = rheumatoid arthritis; wt = wild type.

Genotype analysis revealed the heterozygous presence of CCR5d32 in 23.7% of the healthy controls and 17.3% of the RA patients, while a homozygous CCR5d32 deletion was present in 1.5% of the controls and 1.4% of the RA patients. The distribution of genotypes complied with the Hardy-Weinberg equilibrium, and the differences resulted in a significantly decreased disease susceptibility associated with the presence of the CCR5d32 deletion (OR = 0.67, *P *= 0.0437).

Stratification of RA patients and controls for gender showed an equal distribution of the CCRd32 deletion in both sexes (data not shown). No significant differences in the median age at disease onset (median 49.5 years versus median 49.0 years) or the median disease duration at the time of analysis (median 15.0 years vs. median 16.0 years) were observed between RA patients positive or negative for the CCR5 d32 deletion.

The study cohort analyzed has previously been investigated for an association between a polymorphism in the DNASE2 gene, which codes for an exonuclease required for DNA degradation in lysosomes. Analysis of genotyping results for the CCR5d32 deletion in conjunction with the presence of the homozygous RA associated SNP in the DNASE2 gene revealed an additive effect of the two genetic markers. In patients homozygous for the RA associated DNASE2 -1066 G allele, a further decrease of the frequency of CCR5d32 was statistically significant (10.3% vs. 19.8%, *P *= 0.004 using McNemar's test). Accordingly, the simultaneous presence of homozygosity for DNASE2 -1066 G and of the CCR5 wild type allele was associated with a further increase in RA susceptibility (OR = 2.28, *P *= 0.0051).

Analysis of radiographic findings revealed an association of a milder disease course with the CCR5 d32 deletion (Table 3). In patients without radiographic evidence of bone erosions, the CCR5d32 deletion was present more frequently than in patients with erosive disease (29.6% versus 14.5%, *P *= 0.0047). Accordingly, the increased frequency of the wild type allele in the patient group with erosions resulted in an OR of 2.47 for erosive joint disease. The disease duration at the time of radiographic analysis did not differ between CCR5d32-positive patients with joint erosions and those with non-erosive disease (median 15 years vs. median 13 years, not statistically significant). Furthermore, no significant differences in the patients' age at onset of the disease were found between the two groups (median 56 years vs. median 46 years, not statistically significant).

**Table 3:** Comparison of genotype frequencies for CCR5 d32 in patients with rheumatoid arthritis stratified for erosive disease and extraarticular manifestations

	Genotype		*P *value
		
	wt/wt	wt/d32+d32/d32	
*Erosive disease*			
Yes (n = 296)	253 (85.5)	43 (14.5)	
No (n = 71)	50 (70.4)	21 (29.6)	0.0047
			
*Extraarticular manifestions*			
Yes (n = 144)	125 (86.8)	19 (13.2)	
No (n = 184)	142 (77.2)	42 (22.8)	0.0374

Values are the absolute numbers of genotypes with frequencies in percent given in parentheses. *P *values were calculated using chi-squared test with 2 × 2 contigency tables for comparison of frequencies of the indicated marker in the rheumatoid arthritis patient subgroups. Data for erosive disease status and the presence of extraarticular manifestions was not available for 136 and 175 patients, respectively.

wt = wild type.

**Figure 1:**
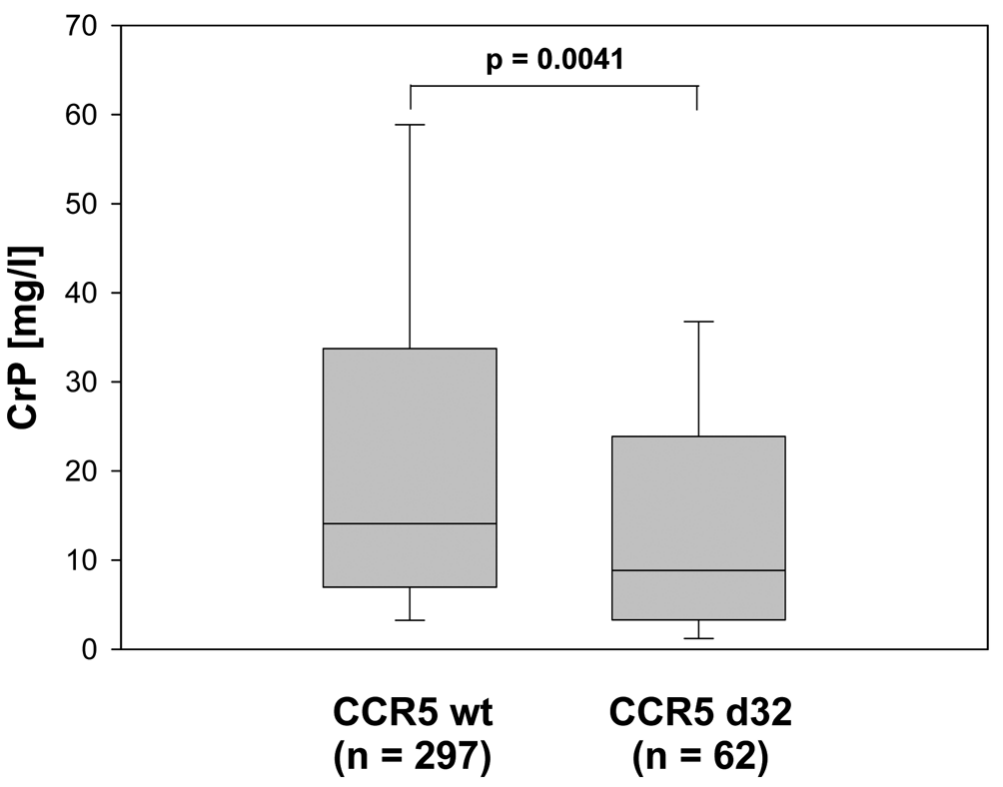
Median CRP levels are lower in patients with rheumatoid arthritis carrying CCR5 d32. Box plot depicts median and interquartile range of the averaged C-reactive protein (CRP) values that were calculated for individual patients from all measurements available for retrospective analysis.

In order to assess the relation between the CCR5d32 deletion and disease activity, all documented CRP values from the patients followed in the Department of Rheumatology at Leipzig University were determined (median 21 values per patient over the total disease duration). Patients carrying the deletion had significantly lower mean CRP values compared with patients homozygous for the wild type (median 8.85 vs. median 14.1, *P *= 0.0041, Figure 1).

To further analyze the influence of the CCR5 d32 deletion on the clinical course of the disease, the occurrence of the following symptoms of extra-articular disease was investigated: presence of nodular RA, major organ vasculitis, serositis, pulmonary fibrosis, or Raynaud's syndrome. The frequency of CCR5 d32 was significantly lower in patients with extra-articular manifestations compared with those without extra-articular disease (13.2% versus 22.8%, *P *= 0.0374). However, no significant association with any individual symptom was discernible, (data not shown).

Stratification of patients for the presence or absence of RF IgM, RF IgA, anti-CCP antibodies, or ANAs did not reveal a significant influence of those parameters on the CCR5 d32 genotype distributions in the patient subgroups (data not shown).

Two independent RA cohorts were chosen for the replication study on the association of CCR5 d32 with the inflammatory response, joint erosion, and DNASE2. In the early RA cohort, CRP levels at the onset of disease were determined. Patients carrying the deletion had significantly lower CRP values at disease onset compared with patients homozygous for the wild type (median 8.0 vs. median 12.0, *P *= 0.028, n = 251).

For both cohorts, radiographic data were available, which had been scored according to the Ratingen score. In the early RA cohort, no significant difference in the Ratingen score between patients carrying the deletion and patients with the wild type CCR5 after two years of disease onset was observed (median 2.0 vs. median 4.0, *P *= 0.5337, n = 141). In the other cohort, radiographic data were available from at least two time points, one taken during the first 10 years of disease duration and one taken after more than 10 years of disease duration. At both time points, a lower Ratingen score was observed in patients with CCR5 d32 in comparison to patients carrying the wild type CCR5 (<10 years: median 7.0 vs. median 10.0, *P *= 0.0324, n = 158; = 10 years: median 14.0 vs. 26.0, *P *= 0.0219, n = 118).

A total of 470 patients of both additional RA cohorts were genotyped for the DNASE2 -1066 SNP. In patients homozygous for the RA associated DNASE2 -1066 G allele, a significant lower frequency of CCR5 d32 was observed (12.5% vs. 22.3%, *P *< 0.0001 using McNemar's test).

## Discussion

We report for the first time a significant influence of the presence of the CCR5 d32 deletion on the inflammatory response in RA, the occurrence of extra-articular manifestations of RA, and on the presence of erosive disease.

Published results of the initial disease association studies are somewhat conflicting. The negative association of the CCR5 d32 deletion with RA susceptibility was observed exclusively for homozygous carriers in one study [5], and was limited to seropositive or more severe disease in other studies [19,20]. More recently, a pooled analysis of all published case-control studies until 2006 [7], and the combination of this meta-analysis with at least one subsequent study [8] supported the notion of a significant negative association of the CCR5 d32 deletion with RA. The data presented here provide further evidence for such a protective effect.

Ethnic effects are likely to account at last in part for some of the discrepancies. The frequency of carriers of the CCR5 d32 deletion varies widely between different ethnic groups [5]. The d32 deletion is absent in native Africans, American Indians, or East Indians, and is likely to have arisen by mutation in northeast Europe, and possibly even from northern Germany [29], which might explain the comparatively high frequency of the allele in our Saxonian, German population. In the initial study cohort, heterozygotes and homozygotes combined account for 25% of healthy controls, which exceeds the frequencies in control populations in many published studies. Therefore, the ethnic characteristic of the German population might contribute to the significant associations observed in the study, because the detection of potential influences of genetic parameters on disease course and susceptibility is facilitated by a higher population frequency.

In addition to the protective effect on disease susceptibility, the CCR5 d32 deletion has previously been reported to influence clinical disease parameters. Patients carrying the deletion allele were preferentially negative for RF IgM, had less frequently swollen joints, and had shorter morning stiffness compared with those patients homozygous for the normal allele [19].

In contrast, a published study in Mexican patients with RA found no difference in allele distribution between RA patients and controls or between treatment refractory and non-refractory patient groups. An ethnic characteristic of this study was the rather low frequency of the CCR5d32 deletion in all analyzed ethnic groups and in the RA patients (<3% of all individuals), which might impede detection of genetic influences with statistical significance [30]. A study by Pokorny and colleagues also detected no influence of the CCR5d32 deletion on disease severity or outcome in the prospective early RA cohort [6]. However, this analysis was performed in an early RA cohort of only 92 patients who were followed prospectively for two years, and significant influences on erosive joint destructions might have been missed due to the small patient number.

In contrast to those earlier reports, we saw no preferential association of the CCR5 d32 deletion with RF seronegative or anti-CCP-negative disease, although there was a trend, which did not reach statistical significance. The strongest influences of the genetic marker observed in our cohort were a significant association with non-erosive joint disease and a decreased inflammatory response in carriers of the deletion. In addition, the partial replication of those clinical associations in two additional study cohorts indicates that the influence of the deletion on the clinical disease course is a relevant and potentially clinically meaningful observation.

We recently reported the association of polymorphisms in the DNASE2 gene, which codes for an exonuclease required for DNA degradation in lysosomes, with increased susceptibility for RA in the same cohort [21]. In addition, DNASE2 knock-out mice spontaneously develop chronic polyarthritis resembling human RA [31]. Here, we report a lower frequency of the CCR5 d32 deletion in RA patients homozygous for the RA associated SNP in the DNASE2 gene and therefore a further increase in RA susceptibility in the simultaneous presence of homozygosity for DNASE2 -1066 G and of the CCR5 wild type allele.

As DNASE2 and CCR5 are both expressed in monocytes and macrophages, one might speculate that increased levels of DNA that have escaped degradation in macrophages due to a lower expression rate of the enzyme might lead to production of chemokines which in turn bind to CCR5 further activating these cells and surrounding cells, such as CD4+ T cells. The expression of non-functional CCR5 in humans carrying the CCR5 d32 deletion would have a protective effect by decreasing the activation of CCR5 and therefore diminishing migration of these cells into the synovial membrane.

The relevance of signaling of CCR5 in destructive arthritis has been demonstrated in primates by the inhibition of collagen-induced arthritis in rhesus monkeys by a CCR5 antagonist [32]. The precise role of CCR5 signaling in the pathogenesis of human RA is unclear, but ligand binding to the receptor has been shown to down-modulate its expression [2], which also appears to be in part genetically determined by the presence of CCR5 d32 [33]. Expression levels of CCR5, in turn, have been reported to be increased in active and decreased in less active disease, and a good clinical response to anti-TNFα treatment might be predicted by high percentages of CCR5 expressing T cells [34]. It could be hypothesized, therefore, that a genetically determined decrease in CCR5 expression is the underlying reason for the observed association with non-erosive and less severe disease.

## Conclusions

In the study presented here, the frequency of a genetic polymorphism resulting in a deletion of the CCR5 gene was analyzed in a large cohort of patients with RA and in healthy controls. The protective effect of the deletion known from other studies could be confirmed. In addition, a significant statistical influence of the polymorphism on the clinical course of the autoimmune disease was observed. Carriers of the deletion were protected from joint erosions, were less frequently affected by extra-articular manifestations of the disease, and had lower cumulative CRP levels. This association indicates clinical usefulness of the deletion as a prognostic diagnostic marker as well as a likely pathogenetic role for CCR5.

## Abbreviations

ANA: antinuclear antibodies; CCP: anti-cyclic citrullinated peptide; CI: confidence interval; CRP: C-reactive protein; PCR: polymerase chain reaction; OR: odds ratio; RA: rheumatoid arthritis; RF: rheumatoid factor.

## Competing interests

The authors declare that they have no competing interests.

## Authors' contributions

MR carried out the molecular genetic studies. MR and MP performed acquisition of the data. MR and CB performed analysis and interpretation of the data. MP, SA and UW contributed to the recruitment of patients and to the acquisition of clinical data. GK and HB contributed the clinical data, radiographic analyses and DNA samples from the patients in the replication studies and were invloved in data analysis. UW drafted the manuscript supported by MR and CB. All authors read and approved the final manuscript.

## References

[B1] Katschke KJ, Rottman JB, Ruth JH, Qin S, Wu L, LaRosa G, Ponath P, Park CC, Pope RM, Koch AE (2001). Differential expression of chemokine receptors on peripheral blood, synovial fluid, and synovial tissue monocytes/macrophages in rheumatoid arthritis. Arthritis Rheum.

[B2] Mack M, Bruhl H, Gruber R, Jaeger C, Cihak J, Eiter V, Plachy J, Stangassinger M, Uhlig K, Schattenkirchner M, Schlondorff D (1999). Predominance of mononuclear cells expressing the chemokine receptor CCR5 in synovial effusions of patients with different forms of arthritis. Arthritis Rheum.

[B3] Samson M, Libert F, Doranz BJ, Rucker J, Liesnard C, Farber CM, Saragosti S, Lapoumeroulie C, Cognaux J, Forceille C, Muyldermans G, Verhofstede C, Burtonboy G, Georges M, Imai T, Rana S, Yi Y, Smyth RJ, Collman RG, Doms RW, Vassart G, Parmentier M (1996). Resistance to HIV-1 infection in caucasian individuals bearing mutant alleles of the CCR-5 chemokine receptor gene. Nature.

[B4] Dean M, Carrington M, Winkler C, Huttley GA, Smith MW, Allikmets R, Goedert JJ, Buchbinder SP, Vittinghoff E, Gomperts E, Donfield S, Vlahov D, Kaslow R, Saah A, Rinaldo C, Detels R, O'Brien SJ (1996). Genetic restriction of HIV-1 infection and progression to AIDS by a deletion allele of the CKR5 structural gene. Hemophilia Growth and Development Study, Multicenter AIDS Cohort Study, Multicenter Hemophilia Cohort Study, San Francisco City Cohort, ALIVE Study. Science.

[B5] Gomez-Reino JJ, Pablos JL, Carreira PE, Santiago B, Serrano L, Vicario JL, Balsa A, Figueroa M, de Juan MD (1999). Association of rheumatoid arthritis with a functional chemokine receptor, CCR5. Arthritis Rheum.

[B6] Pokorny V, McQueen F, Yeoman S, Merriman M, Merriman A, Harrison A, Highton J, McLean L (2005). Evidence for negative association of the chemokine receptor CCR5 d32 polymorphism with rheumatoid arthritis. Ann Rheum Dis.

[B7] Prahalad S (2006). Negative association between the chemokine receptor CCR5-Delta32 polymorphism and rheumatoid arthritis: a meta-analysis. Genes Immun.

[B8] Lindner E, Nordang GB, Melum E, Flato B, Selvaag AM, Thorsby E, Kvien TK, Forre OT, Lie BA (2007). Lack of association between the chemokine receptor 5 polymorphism CCR5delta32 in rheumatoid arthritis and juvenile idiopathic arthritis. BMC Med Genet.

[B9] McKinney C, Merriman ME, Chapman PT, Gow PJ, Harrison AA, Highton J, Jones PB, McLean L, O'Donnell JL, Pokorny V, Spellerberg M, Stamp LK, Willis J, Steer S, Merriman TR (2008). Evidence for an influence of chemokine ligand 3-like 1 (CCL3L1) gene copy number on susceptibility to rheumatoid arthritis. Ann Rheum Dis.

[B10] Kantarci OH, Morales Y, Ziemer PA, Hebrink DD, Mahad DJ, Atkinson EJ, Achenbach SJ, De Andrade M, Mack M, Ransohoff RM, Lassmann H, Bruck W, Weinshenker BG, Lucchinetti CF (2005). CCR5Delta32 polymorphism effects on CCR5 expression, patterns of immunopathology and disease course in multiple sclerosis. J Neuroimmunol.

[B11] Mamtani M, Rovin B, Brey R, Camargo JF, Kulkarni H, Herrera M, Correa P, Holliday S, Anaya JM, Ahuja SK (2008). CCL3L1 gene-containing segmental duplications and polymorphisms in CCR5 affect risk of systemic lupus erythaematosus. Ann Rheum Dis.

[B12] Herfarth H, Pollok-Kopp B, Goke M, Press A, Oppermann M (2001). Polymorphism of CC chemokine receptors CCR2 and CCR5 in Crohn's disease. Immunol Lett.

[B13] Petrek M, Cermakova Z, Hutyrova B, Micekova D, Drabek J, Rovensky J, Bosak V (2002). CC chemokine receptor 5 and interleukin-1 receptor antagonist gene polymorphisms in patients with primary Sjogren's syndrome. Clin Exp Rheumatol.

[B14] Yang X, Ahmad T, Gogus F, Verity D, Wallace GR, Madanat W, Kanawati CA, Stanford MR, Fortune F, Jewell DP, Marshall SE (2004). Analysis of the CC chemokine receptor 5 (CCR5) Delta32 polymorphism in Behcet's disease. Eur J Immunogenet.

[B15] Spagnolo P, Renzoni EA, Wells AU, Copley SJ, Desai SR, Sato H, Grutters JC, Abdallah A, Taegtmeyer A, du Bois RM, Welsh KI (2005). C-C chemokine receptor 5 gene variants in relation to lung disease in sarcoidosis. Am J Respir Crit Care Med.

[B16] Henckaerts L, Fevery J, Van Steenbergen W, Verslype C, Yap P, Nevens F, Roskams T, Libbrecht L, Rutgeerts P, Vermeire S (2006). CC-type chemokine receptor 5-Delta32 mutation protects against primary sclerosing cholangitis. Inflamm Bowel Dis.

[B17] Afzal AR, Kiechl S, Daryani YP, Weerasinghe A, Zhang Y, Reindl M, Mayr A, Weger S, Xu Q, Willeit J (2008). Common CCR5-del32 frameshift mutation associated with serum levels of inflammatory markers and cardiovascular disease risk in the Bruneck population. Stroke.

[B18] Prahalad S, Bohnsack JF, Jorde LB, Whiting A, Clifford B, Dunn D, Weiss R, Moroldo M, Thompson SD, Glass DN, Bamshad MJ (2006). Association of two functional polymorphisms in the CCR5 gene with juvenile rheumatoid arthritis. Genes Immun.

[B19] Garred P, Madsen HO, Petersen J, Marquart H, Hansen TM, Freiesleben Sorensen S, Volck B, Svejgaard A, Andersen V (1998). CC chemokine receptor 5 polymorphism in rheumatoid arthritis. J Rheumatol.

[B20] Zapico I, Coto E, Rodriguez A, Alvarez C, Torre JC, Alvarez V (2000). CCR5 (chemokine receptor-5) DNA-polymorphism influences the severity of rheumatoid arthritis. Genes Immun.

[B21] Rossol M, Pierer M, Arnold S, Keyszer G, Burkhardt H, Baerwald C, Wagner U (2008). Homozygosity for DNASE2 single-nucleotide polymorphisms in the 5' regulatory region is associated with rheumatoid arthritis. Ann Rheum Dis.

[B22] Pierer M, Kaltenhauser S, Arnold S, Wahle M, Baerwald C, Hantzschel H, Wagner U (2006). Association of PTPN22 1858 single-nucleotide polymorphism with rheumatoid arthritis in a German cohort: higher frequency of the risk allele in male compared to female patients. Arthritis Res Ther.

[B23] Malysheva O, Pierer M, Wagner U, Wahle M, Baerwald CG (2008). Association between beta2 adrenergic receptor polymorphisms and rheumatoid arthritis in conjunction with human leukocyte antigen (HLA)-DRB1 shared epitope. Ann Rheum Dis.

[B24] Wagner U, Pierer M, Kaltenhauser S, Wilke B, Seidel W, Arnold S, Hantzschel H (2003). Clonally expanded CD4+CD28null T cells in rheumatoid arthritis use distinct combinations of T cell receptor BV and BJ elements. Eur J Immunol.

[B25] Dorr S, Lechtenbohmer N, Rau R, Herborn G, Wagner U, Muller-Myhsok B, Hansmann I, Keyszer G (2004). Association of a specific haplotype across the genes MMP1 and MMP3 with radiographic joint destruction in rheumatoid arthritis. Arthritis Res Ther.

[B26] Burkhardt H, Huffmeier U, Spriewald B, Bohm B, Rau R, Kallert S, Engstrom A, Holmdahl R, Reis A (2006). Association between protein tyrosine phosphatase 22 variant R620W in conjunction with the HLA-DRB1 shared epitope and humoral autoimmunity to an immunodominant epitope of cartilage-specific type II collagen in early rheumatoid arthritis. Arthritis Rheum.

[B27] Huffmeier U, Boiers U, Lascorz J, Reis A, Burkhardt H (2008). Loss-of-function mutations in the filaggrin gene: no contribution to disease susceptibility, but to autoantibody formation against citrullinated peptides in early rheumatoid arthritis. Ann Rheum Dis.

[B28] Rau R, Wassenberg S, Herborn G, Stucki G, Gebler A (1998). A new method of scoring radiographic change in rheumatoid arthritis. J Rheumatol.

[B29] Novembre J, Galvani AP, Slatkin M (2005). The geographic spread of the CCR5 Delta32 HIV-resistance allele. PLoS Biol.

[B30] Zuniga JA, Villarreal-Garza C, Flores E, Barquera R, Perez-Hernandez N, Montes de Oca JV, Cardiel MH, Vargas-Alarcon G, Granados J (2003). Biological relevance of the polymorphism in the CCR5 gene in refractory and non-refractory rheumatoid arthritis in Mexicans. Clin Exp Rheumatol.

[B31] Kawane K, Ohtani M, Miwa K, Kizawa T, Kanbara Y, Yoshioka Y, Yoshikawa H, Nagata S (2006). Chronic polyarthritis caused by mammalian DNA that escapes from degradation in macrophages. Nature.

[B32] Vierboom MP, Zavodny PJ, Chou CC, Tagat JR, Pugliese-Sivo C, Strizki J, Steensma RW, McCombie SW, Celebi-Paul L, Remarque E, Jonker M, Narula SK, Hart B (2005). Inhibition of the development of collagen-induced arthritis in rhesus monkeys by a small molecular weight antagonist of CCR5. Arthritis Rheum.

[B33] Wu L, Paxton WA, Kassam N, Ruffing N, Rottman JB, Sullivan N, Choe H, Sodroski J, Newman W, Koup RA, Mackay CR (1997). CCR5 levels and expression pattern correlate with infectability by macrophage-tropic HIV-1, in vitro. J Exp Med.

[B34] Nissinen R, Leirisalo-Repo M, Peltomaa R, Palosuo T, Vaarala O (2004). Cytokine and chemokine receptor profile of peripheral blood mononuclear cells during treatment with infliximab in patients with active rheumatoid arthritis. Ann Rheum Dis.

